# Coconut rhinoceros beetle, *Oryctes rhinoceros* (Coleoptera: Scarabaeidae), larval frass as plant fertilizer

**DOI:** 10.1186/s40529-025-00459-x

**Published:** 2025-08-05

**Authors:** Chiao-Jung Han, Zeng-Yei Hseu, Po-Hui Wu, Louis Grillet, Chun-Han Ko, Matan Shelomi

**Affiliations:** 1 Department of Entomology, National Taiwan University, Taipei, Taiwan; 2 Institute of Plant and Microbial Biology, Academia Sinica, Taipei, Taiwan; 3 Department of Agricultural Chemistry, National Taiwan University, Taipei, Taiwan; 4 School of Forestry & Resource Conservation, National Taiwan University, Taipei, Taiwan

**Keywords:** Elemental analysis, Fertilizer, Frass, Microbiome, Rhinoceros beetle, Soil amendment

## Abstract

**Background:**

Beetle rearing for food or feed is a growing area of agriculture that produces considerable wastes. This frass is a putative soil amendment anecdotally applied directly as fertilizer. To determine if beetle waste can be used as a soil amendment without pre-treatment, a series of chemical, physical, microbiological, and plant-growth assays were performed on waste from the coconut rhinoceros beetle, *Oryctes rhinoceros* (Coleoptera: Scarabaeidae), fed cocopeat. Beetle diet and frass NPK levels, C:N ratio, and particle size were measured. Microbiota was identified with metabarcoding, and functional profile analysis done to identify pathways associated with wood digestion or plant growth. Cultivation tests were done with *Arabidopsis thaliana* (Brassicales: Brassicaceae) and frass incorporation into potting soil at 0, 20, or 40%, followed by elemental concentration measurement of the soil, frass, and plant matter.

**Results:**

Digestion of plant polysaccharides in the gut, primarily by microbial depolymerizers, produces frass of uniform particle size and NPK 1.8–0.13–1.2 that can be used directly as a mature fertilizer without pre-composting, or even as a growth substrate. Plants with higher proportions of frass in the soil grew significantly faster and larger compared to a nutrient-rich potting soil. Frass is high in useful elements and has beneficial chitinolytic microbes.

**Conclusions:**

Beetle frass can thus be used directly as a soil amendment without composting or pretreatment, with positive effects on plant growth even compared to rich soils. Valorizing frass in this way generates income for beetle farmers and recycles nutrients to soil as part of circular agriculture.

**Supplementary Information:**

The online version contains supplementary material available at 10.1186/s40529-025-00459-x.

## Background

Animal manure has a long history of use as fertilizer, including invertebrate manure such as earthworm castings. Recent data suggests the frass of farmed insects has high fertilizing index values, capable of enhancing plant defenses under biotic or abiotic stress and providing more plant accessible nutrients (Lovett and Ruesink 1995; Barragán-Fonseca et al. 2022; Blakstad et al. 2023; Poveda et al. 2019; Watson et al. 2021), although some species’ frass requires prior composting to mature (Beesigamukama et al. 2022). These studies have been predominantly on frass from the three most commonly farmed insects (Davidowitz 2021): mealworms (Blakstad et al. 2023; Poveda et al. 2019; Wantulla et al. 2022), black soldier fly (Wantulla et al. 2022; Wang et al. 2022; Lopes et al. 2022), and house cricket (Wantulla et al. 2022). However, other species are or can be farmed more sustainably in certain habitats than these (Davidowitz 2021). Their frass too warrants investigation, as valorizing this waste could potentially bring more profits to their farmers, especially small-scale and developing world farmers (Beesigamukama et al. 2022).

Among the beetles (Coleoptera), several species besides mealworms (Tenebrionidae), are already reared commercially. This includes the palm weevil larvae (Curculionidae) reared as food in the tropics (Cito et al. 2017), and the long-horned (Cerambycidae), rhinoceros (Scarabaeidae: Dynastinae), and stag beetles (Lucanidae) reared in East Asia for trade and as pets. Beetles can produce prodigious amounts of waste: the frass of leaf-defoliating beetles was found to contribute significant amounts of nitrogen to the soil and lead to greater nitrogen levels in plant tissues (Uriarte 2000; Gherlenda et al. 2016). Informally, beetle breeders do give their beetle frass to farmers for use as fertilizer. However, with the exception of mealworm frass, the potential risks and benefits of using beetle frass as a soil amendment have not been rigorously analyzed. In fact, only one publication has looked at other beetles’ frass, specifically the African fruit beetle (*Pachnoda sinuata* L.) and the coconut rhinoceros beetle (*Oryctes rhinoceros* L.); both are scarab beetles whose frass showed promising results (Beesigamukama et al. 2022).

This study sought to determine with greater detail the prospects for *Oryctes rhinoceros* frass use as a soil amendment or fertilizer. The species was chosen as it is a pest on palm trees (Pradipta et al. 2020) that nonetheless has potential as a food (Bolaji et al. 2021) and frass source (Beesigamukama et al. 2022), and is an easy-to-rear, inexpensive model for the Scarabaeidae beetles. Open questions about the frass include the impact of its microbiome on plant growth. Insect frass can transmit microbes the insects obtained from their diet, which could include plant pathogens as well as beneficial microbes (Poveda 2021; Osimani et al. 2018). Other potential effects of the microbiome include phytohormone synthesis, nitrogen fixation, phosphorus solubilization, sulfur oxidation, and plant cell wall depolymerization. The *O. rhinoceros* larval gut contains a complex microbiome capable of digesting wood polysaccharides such as cellulose, hemicellulose, and lignin (Han et al. 2024). The effects of these microbes in the frass when applied as a plant fertilizer are unknown.

Phytotoxicity from application of immature compost is another issue. The prior study on *Oryctes rhinoceros* and other insects’ frasses as a soil amendment found the frasses had sufficient values for indices like pH, C/N ratio, and ammonium/nitrate ratio to qualify as a mature compost, but the *O. rhinoceros* frass had moderate phytotoxicity and “minimal suitability for crop production” (Beesigamukama et al. 2022). However, anecdotal use of *O. rhinoceros* frass as a fertilizer suggests that conclusion is not universal.

The goals of this study were to characterize *O. rhinoceros* frass, determine what nutrients it could provide to plants, what microbes it contains, and whether it can be used directly as a soil amendment without pre-treatment. We hypothesized based on its use by farmers that there would be minimal to no phytotoxicity from the frass, and that its nutrient quality and/or microbial components could provide measurable benefits to plants grown in soil with added frass.

## Materials and methods

### Beetle rearing

*Oryctes rhinoceros* beetles collected from decaying palm logs in southern Taiwan were maintained in the Department of Entomology laboratory for one generation on a diet of commercial cocopeat (*Cocos nucifera*, Arecales: Arecaecae) (AgroBrothers Coir Company, India) supplemented with shredded areca palm wood (*Areca catechu*, Arecales: Arecaecae). The frass from third instar larvae was collected by sifting through a sieve, and stored in a sealed plastic bag to reduce loss of nitrogen in a refrigerator to prevent decay. Approximately 500 g of frass could be produced by ten beetle larvae over the course of one week.

### Chemical and physical analysis

The effects of beetle biocomposting on nutrient concentrations were investigated. Samples of beetle frass, commercial cocopeat, and the shredded wood were homogenized and analyzed, including NPK levels and C:N ratio. All air-dried samples were ground to pass through a 20 mesh sieve, heated at 70 °C for 24 h to inhibit enzyme activity, and stored in plastic bags prior to analysis. To determine the moisture content, two grams of samples were oven-heated at 105 °C for 5 h and weighed. The mineral content was determined as followed. For dry ashing, two grams of samples in crucibles were placed in a furnace at 250 °C for 1 h, and then at 550 °C for 4 h, after which the samples were removed from the furnace and cooled down. The samples were weighed and placed in the furnace again until their weight remained constant. After calculating the percentage of moisture and minerals, the sum of the two were subtracted from 100% to get deduct the percentage of organic matter (Nelson and Sommers 1982). The total nitrogen content was determined by the Kjeldahl method (Bremner 1960). The pH of the sample was determined by a LAQUA F-71 glass electrode (HORIBA Advanced techno Co., Ltd.) with the ratio of sample/water in 1:5 (McLean 1982). The total contents of P, K, Fe, Mn, Cu, and Zn, were measured by portable x-ray fluorescence (pXRF) using an Olympus DP-2000-*C* Delta Premium Alloy XRF Analyzer. Statistical analysis was performed using one way ANOVA followed by a post hoc Tukey HSD Test using an online calculator (https://astatsa.com/).

Particle size distribution and shape analysis was used to observe the impact of beetle larvae digestion on the physical properties of the cocopeat (Holzinger et al. 2023). For this, 100 g of cocopeat and 100 g of frass dissolved in water and sonicated into separate particles were analyzed using a Microtrac Flow Synchronous Laser Diffraction machine (Microtrac MRB, Montgomeryville, PA, USA) and Dynamic Image Particle Analyzer (Microtrac MRB, Montgomeryville, PA, USA).

### Microbiome analysis

As soil or compost microbiota can stimulate plants to make a beneficial lignin barrier on the roots, microbial analysis was performed. Three biological replicates each were sampled from the lived-in larval rearing substrate or “beetle soil,” the cocopeat direct from the package, larval fecal pellets taken from the larval containers [“old frass”], and fresh larval fecal pellets coaxed directly from the larvae into a sterile collection tube [“fresh frass”]. Their DNA was extracted using a PrestoTM Soil DNA Extraction Kit (Geneaid) according to the manufacturer’s instructions. The extracted DNA was pre-purified using the OneStep PCR Inhibitor Removal Kit (Zymo Research) and then purified with a DNeasy® PowerClean® Pro Cleanup Kit (QIAGEN). The purified DNA samples were sent to BioTools Co. Ltd. (Taiwan) for amplification sequencing of the hypervariable V3 and V4 regions of the 16S rDNA using paired-end Illumina Miseq (300 bp paired-end reads). The sequencing data have been deposited in NCBI GenBank (Bioproject: PRJNA1052367). Following sequencing, QIIME2 v2023.9 (Bolyen et al. 2019; Estaki et al. 2020) was used with default parameters for quality filtering and microbiome analysis as reported previously (Shelomi and Chen 2020). Alpha diversity was measured as Shannon, Faith PD, and Pielou’s evenness with Kruskal–Wallis and pairwise Kruskal–Wallis tests using QIIME2. Beta diversity by Bray–Curtis was compared with Permanova and pairwise Adonis tests using the *vegan, pairwiseAdonis,* and *ggplot2* packages in R (Oksanen 2017). An NCBI RefSeq classifier for prokaryotic 16S *rRNA* (33175[Bioproject] OR 33317[Bioproject]) was generated using RESCRIPt (Robeson et al. 2021; O’Leary et al. 2016; Tatusova et al. 2016) for the taxonomic assignments. Functional profile analysis was used to see the potential bio-pathways or functions provided by microbes in the *O. rhinoceros* frass. This was done using the standard pipeline of PICRUSt2 v2.4.1 (Douglas et al. 2020) on the online Nephele:Microbiome web server of U.S. National Institutes of Health & National Institute of Allergy & Infectious Diseases (https://nephele.niaid.nih.gov/init_picrust2) for KEGG orthology prediction of genes associated with the following: synthesis of plant growth hormone (auxins and cytokinins); nitrogen fixation, phosphorus solubilization, and sulfur oxidation pathways; and plant cell wall and exoskeleton depolymerization (plant cell wall degrading enzymes and chitinases) (Table 1). To investigate microbial composition differences among the four sample types (cocopeat, beetle soil, fresh frass collected directly from the larva, old frass collected from the substrate), a non-metric multidimensional scaling (nMDS) plot was utilized to visually represent clustering patterns, along with corresponding statistical analyses.

**Table 1:** The functional groups with enzymes/genes for PICRUSt2 analysis

Functional group	Enzymes/genes were searched
Auxin synthesis	Amine-oxidase, tryptophan transaminase, *dhaS, iaaH,* IAAId dehydrogenase, *iaaM*, IAM-hydrolase, *IPdC*, nitrilase, nitrilehydratase, *pat*B, trp decarboxylase, *trpA, trpB, trpD*, *trpE,* tryptophan side-chain oxidase, *yclB, yclC, yhcX, ysnE* (Keswani et al. 2020; Spaepen and Vanderleyden 2011)
Cytokinin synthesis	Beta-glucosidase, *CKX, CYP450, FasD, iPRMP, ipt, LAS, LDCs, LOG, MiaB, MiaE, N-GT, NoIPT1, Tmr, tZRMP,Tzs, ZI, ZOGT, ZR* (Frébort et al. 2011; Frébortová and Frebort 2021; Seo et al. 2016)
Nitrogen fixation	*amtB, AnfG, draG, draT, fixA, fixB, fixC, fixJ, fixK, fixL, fixL-fixJ, fixR, fixT, fixX, glnB, glnK, nifA, nifB, nifD,nifE, nifH, nifK, nifL, nifN, nifP,nifQ,nifS, nifT, nifU, nifV, nifW, nifX, nifZ, nrf1,NtrB, NtrB-NtrC, NtrC, RegB, RegR, RegS-RegR, VnfG* (Dixon and Kahn 2004; Santos et al. 2012; Dai et al. 2014)
Phosphorus solubilization	*acpA, aphA, appA, appA2, gabY, gcd, mps, napA, napD, napE, olpA, pcc, phnC, phnD, phnE, phnG, phnH, phnI, phnJ, phnL, phnM, phnN, phnO, phnP, phnW, phnX, phoA, phoB, PhoC, phoD, phoN, phoR, phoX, phy, phyA, ppa, ppx, pstA, pstB, pstC, pstS, ugpA, ugpB, ugpC, ugpE, ugpQ* (Rodríguez et al. 2006; Liang et al. 2020)
Sulfur oxidation	*APR, AprBA, DoxDA, DsrA, DsrB, DsrC, DsrE, DsrE2B, DsrE3, DsrE3A, DsrEFH, DsrF, DsrH, DsrJ, DsrK,DsrM, DsrO, DsrP, FCC, Hdr,Rhd, SAT, SDH, SoxB, SoxCD, SoxXA, SoxY, SoxYZ, SoxYZXA, SoxZ, SQR, TcDH, Tqo, TusA* (Dahl 2015; Friedrich et al. 2005)
Cell wall and exoskeleton depolymerization	Plant cell wall degrading enzymes as recorded on dbCAN2 web server (https://bcb.unl.edu/dbCAN2/) (Zhang et al. 2018) including those from auxiliary activities, carbohydrate esterase, glycosidase hydrolase, polysaccharide lyase families, etc
Chitin degradation	As recorded on dbCAN2 web server (Zhang et al. 2018)

#### Cultivation test and determination of elemental concentrations

To determine the effect of fertilization with beetle frass on plant growth, the standard laboratory model plant *Arabidopsis thaliana* (L.) Heynh accession Columbia (Col-0) (Somssich 2019) (Brassicales: Brassicaceae) was grown in a climate chamber at 22 °C (16 h light/8 h dark). Plants were irrigated once a week with demineralized water. The soil consisted of a mix of four parts of potting soil (Jiffy®, The Netherlands), one part of perlite, and one part of vermiculite; and fresh frass was incorporated or not to the mix at 0%, 20%, or 40% w/w. The same ratios or higher (up to 50%) have been used in published studies of insect frass as a soil amendment (Tan et al. 2021; Song et al. 2021; Houben et al. 2020). One replicate consisted of six pots of each treatment, and the 18 pots of each experiment were placed randomly in a 10 ×20-inch tray. The experiment was carried out in three independent replicates. When the last plant started flowering, which happened at seven weeks after sowing, the height of the inflorescences was measured, the plants were harvested, and their fresh and dry weight was recorded individually. Statistical analysis was done by with a one way ANOVA followed by a Duncan test with *p* < 0.05 using R’s *agricolae* package (de Mendiburu and Yaseen 2020).

To measure the elemental concentrations in the plant tissues, the harvested plants were washed twice in 10 mM CaCl_2_ and 1 mM EDTA and twice with ultrapure water. Each batch of six plants were dried in an oven at 60 °C for 72 h. Ten mg of dry tissues were mineralized in 65% HNO_3_ in a microwave, homogenized with mortar and pestles, and diluted in 10 mL ultrapure water before analysis with an Agilent 7800 ICP-MS. A certified sample of tomato leaves was also analyzed to evaluate the accuracy. Each sample was processed and analyzed in duplicate. Statistical analysis was done by with a one way ANOVA followed by a Duncan test with *p* < 0.05 using R’s *agricolae* package (de Mendiburu and Yaseen 2020).

An elemental concentration test was also done to compare the potting soil and the frass. For each, a 0.2 g sample was mixed with 6 mL of HNO₃ in a Teflon vessel and left to react for 14 h. The sample was then pre-digested by heating to 100 °C over 10 min in a microwave oven (Speedwave Entry, Berghof, Eningen, Germany). Subsequently, 2 mL of 35% H₂O₂ was added, and the temperature was gradually increased to 120 °C over eight minutes, where it was held for two minutes. The temperature was further raised to 160 °C within five minutes and maintained for another five minutes. In the final stage, it was increased to 180 °C over five minutes and held for 15 min. After cooling, the digested solution was diluted to 10 mL with 2% HNO₃ and filtered through a 0.45 μm Millipore filter. The elemental concentrations for Al, B, Ca, Cr, Cu, K, Mg, Mn, Na, and P were determined by inductively coupled plasma optical emission spectroscopy (ICP-OES, Optima 2100DV, PerkinElmer, Waltham, MA, USA), and the rest with pXRF as described above. Means for frass and soil were compared using a one-tailed, unpaired t-test.

## Results

### Chemical and physical analyses of beetle diet and frass

The results of the nutrient analyses are in Table 2. The beetle’s diet was comprised mostly of cocopeat with areca wood supplementation. No significant differences were found between the levels in the frass and the cocopeat of nitrogen, potassium, copper, and ash. Compared to the initial wood, the beetle frass contained more nitrogen, phosphorus, potassium, iron, and manganese per unit weight, and less carbon, copper, and zinc (Table 2). The pH of the frass was 7.6, compared to the acidic wood at 5.7. The commercial cocopeat’s values were in between those of the frass and the wood, except they were higher in phosphorus, potassium, and manganese and lower in zinc, with a pH of 7.8. Whereas the initial wood pulp had an NPK level of approximately 0.9–0.07–0.8 and the cocopeat an NPK of 1.5–0.2–1.3, the frass has an NPK of approximately 1.8–0.13–1.2. The C:N ratio of the frass was the lowest with a mean of 22.8. Supplementary Fig. 1 shows the distribution for the cocopeat particles, which is discreet and noncontinuous as it comes from shredded plant parts, and for the insect frass, which is uniformly and continuously distributed with significantly smaller particles.

**Table 2:** Inorganic and organic chemical composition of areca nut stalk wood, commercial cocopeat, and the frass from coconut rhinoceros beetles fed this wood

	Insect frass	Areca wood	Cocopeat	*F*_*(2)*_ and *p*-value
N (g/kg)	17.7 ± 1.4^a^	8.8 ± 1.2^b^	14.9 ± 0.4^a^	33.56**
P (g/kg)	1.28 ± 0.07^b^	0.73 ± 0.06^c^	2.13 ± 0.06^a^	261.8***
K (g/kg)	12.0 ± 1.0^a^	7.8 ± 0.2^b^	12.6 ± 1.1^a^	17.7*
Fe (g/kg)	3.03 ± 0.07^a^	0.82 ± 0.06^c^	2.08 ± 0.10^b^	409.7***
Mn (mg/kg)	349 ± 5.7^a^	122 ± 4.2^c^	281 ± 4.2^b^	1197***
Cu (mg/kg)	16 ± 1.7^b^	33 ± 2.4^a^	11 ± 1.5^b^	72.4**
Zn (mg/kg)	103 ± 5.4^b^	165 ± 4.2^a^	81 ± 3.1^c^	198.4***
pH	7.6 ± 0^b^	5.7 ± 0^c^	7.85 ± 0.07^a^	1659***
Average % Moisture	11.9 ± 0.06^a^	9.4 ± 0.1^b^	10.8 ± 0.1^c^	318.4***
Average % ash	18.5 ± 0.2^a^	3.26 ± 0.09^b^	17.9 ± 0.23^a^	3518***
% Organic Matter	69.6 ± 0.3^c^	87.3 ± 0.2^b^	71.3 ± 0.2^a^	3969***
% N	1.78 ± 0.14^a^	0.88 ± 0.12^b^	1.49 ± 0.04^a^	33.56**
% Organic C	40.4 ± 0.2^c^	50.6 ± 0.1^a^	41.4 ± 0.1^b^	6415***
NPK	1.8–0.13–1.2	0.9–0.07–0.8	1.5–0.2–1.3	
C:N ratio	22.8 ± 2.0^b^	58.1 ± 8.1^a^	27.8 ± 0.9^b^	31.1**

Data presented is averages of two samples ± standard deviation. pH was measured in samples dissolved 1:5 in water. Values followed by different superscript letters are statistically different from each other, as determined by a post hoc Tukey HSD test with a *p*-value limit of 0.05 following one-way ANOVA, for which the *F* value (df = 2) is given with *p* values depicted as follows: • =  ≤ 0.1, * =  ≤ 0.05, ** =  ≤ 0.01 *** =  < 0.001.

### Microbiome analysis

**Fig. 1:**
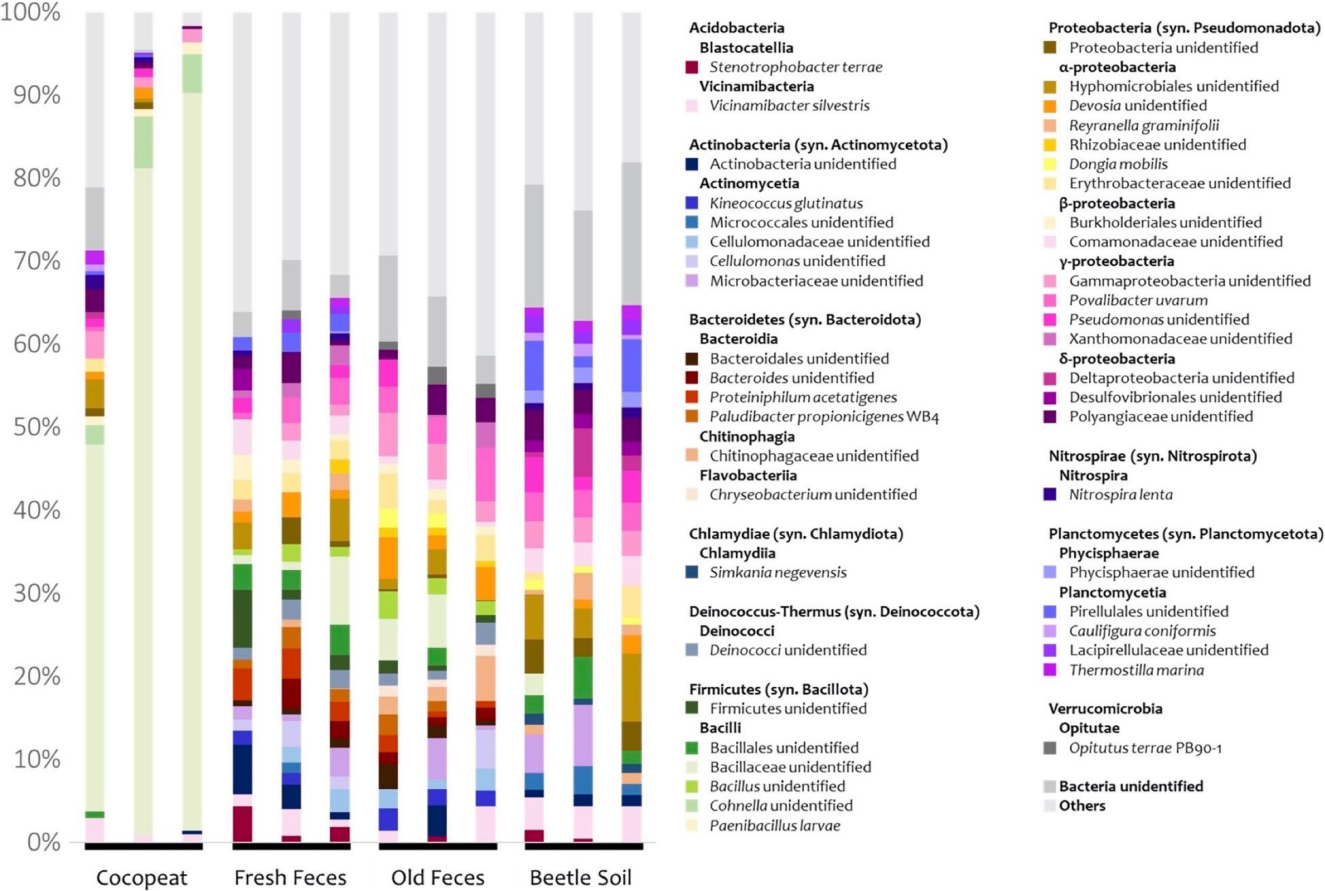
Microbe relative abundance of *Oryctes rhinoceros* frass and diet by taxonomic composition

The 16S V3 and V4 sequences representing microbiomes from the food sources and frass contained 49,550 high-quality and filtered sequences, and produced 308 amplicon sequence variants (ASVs) with a median of 4272 reads per sample and 66 ASVs per sample. A total of one kingdom (Bacteria), 13 phyla, 27 classes, 39 orders, 58 families, 61 genera, and 41 species were classified from the total ASVs. The phyla Firmicutes (syn. Bacillota), Proteobacteria (syn. Pseudomonadota), Actinobacteria (syn. Actinomycetota), Bacteroidetes (syn. Bacteroidota), and Planctomycetes (syn. Planctomycetota), were the five most abundant (Fig. 1). There was only one 100% shared microbe among every sample, identified as a member of Polyangiaceae (Deltaproteobacteria, syn. Myxococcota) (Fig. 1 & Table 3).

**Table 3:** Frequency of occurrence of each microbial clade among cocopeat, fresh frass, old frass, and beetle soil, with its potential functions

Assigned taxonomy	Potential functions associated with plants based on literature	Cocopeat	Fresh frass	Old frass	Beetle soil
Acidobacteriota (syn. Acidobacteria)					
*Stenotrophobacter terrae*	PGPR (Sato et al. 2023)	$\square$	■	$\square$	$\square$
Vicinamibacter silvestris	Alleviating the accumulation of heavy metals in plants (Yu et al. 2023)	■	■	$\square$	■
Actinomycetota (syn. Actinobacteria)					
Micrococcales unidentified	NA	$\square$	$\square$	$\square$	■
Cellulomonadaceae unidentified	Cellulolytic/lignocellulytic (Yeager et al. 2017)	$\square$	$\square$	■	$\square$
Microbacteriaceae unidentified	Cellulolytic (Bashir et al. 2013)	$\square$	■	$\square$	$\square$
Cellulomonas unidentified	Cellulolytic/lignocellulytic (Yeager et al. 2017; Poulsen et al. 2016)	$\square$	■	$\square$	$\square$
Kineococcus glutinatus	NA	$\square$	$\square$	■	$\square$
Bacteroidota (syn. Bacteroidetes)					
Bacteroidales unidentified	Cellulolytic (Naas et al. 2015)	$\square$	■	■	$\square$
Chitinophagaceae unidentified	Denitrification (Ni et al. 2023), PGPR (Madhaiyan et al. 2015)	$\square$	$\square$	■	$\square$
Bacteroides unidentified	Cellulolytic/xylanolytic (Daniel et al. 1995; Forsberg et al. 1981)	$\square$	$\square$	■	$\square$
Chryseobacterium unidentified	Phosphate solubilization, PGPR (Singh et al. 2013)	$\square$	$\square$	■	$\square$
Paludibacter propionicigenes WB4	Nitrogen fixation (Ceja-Navarro et al. 2014; Tai et al. 2016; Sapountzis et al. 2016)	$\square$	■	$\square$	$\square$
Proteiniphilum acetatigenes	Cellulolytic, hemicellulolytic, ligninolytic (Thongbunrod and Chaiprasert 2023)	$\square$	■	■	$\square$
Chlamydiota (syn. Chlamydiae)					
Simkania negevensis	NA	$\square$	$\square$	$\square$	■
Deinococcota (syn. Deinococcus-Thermus)					
Deinococci unidentified	Alleviating the accumulation of heavy metals in plants (Dai et al. 2021)	$\square$	■	■	$\square$
Bacillota (syn. Firmicutes)					
Bacillales unidentified	PGPR, nitrogen fixation	$\square$	■	$\square$	■
Bacillaceae unidentified	PGPR, nitrogen fixation (Mandic-Mulec et al. 2015)	■	■	$\square$	$\square$
Bacillus unidentified	PGPR (Sansinenea 2019), nitrogen fixation (Mandic-Mulec et al. 2015)	$\square$	■	■	$\square$
Cohnella unidentified	Xylanolytic (Hameed et al. 2013; Pisa et al. 2022)	■	$\square$	$\square$	$\square$
Paenibacillus larvae	PGPR, nitrogen fixation, phosphate solubilization, production of IAA, release of siderophores (Grady et al. 2016)	■	$\square$	$\square$	$\square$
Pseudomonadota (syn. Proteobacteria)					
Burkholderiales unidentified	PGPR (Kurepin et al. 2014)	$\square$	■	■	$\square$
Desulfovibrionales unidentified	Nitrogen fixation (Sayavedra et al. 2021)	$\square$	$\square$	$\square$	■
Gammaproteobacteria unidentified	PGPR (Kumar Ghosh et al. 2015; Kuan et al. 2016)	■	$\square$	■	■
Deltaproteobacteria unidentified	Nitrogen fixation (Langwig et al. 2022)	$\square$	$\square$	$\square$	■
Hyphomicrobiales unidentified	Nitrogen fixation (Im et al. 2006; Wiegel et al. 1978)	$\square$	$\square$	$\square$	■
Comamonadaceae unidentified	Sulfur fertilization (Bao and Li 2017; Schmalenberger et al. 2008), nitrification and denitrification (Bao and Li 2017)	$\square$	■	■	■
Erythrobacteraceae unidentified	NA	$\square$	■	■	$\square$
Polyangiaceae unidentified	NA	■	■	■	■
Rhizobiaceae unidentified	Nitrogen fixation (Lindstrom and Mousavi 2020)	$\square$	$\square$	■	$\square$
Xanthomonadaceae unidentified	Pectinolytic (Vorhölter et al. 2012)	$\square$	■	$\square$	$\square$
Devosia unidentified	Nitrogen fixation (Rivas et al. 2002), PGPR (Chhetri et al. 2023), Bioremediation (Talwar et al. 2020)	$\square$	■	■	$\square$
Dongia mobilis	NA	$\square$	$\square$	$\square$	■
Povalibacter uvarum	NA	$\square$	■	■	■
Pseudomonas unidentified	PGPR (Vorhölter et al. 2012; Preston 2004; Mercado-Blanco and Bakker 2007)	$\square$	$\square$	$\square$	■
Reyranella graminifolii	Nitrogen fixation (Li et al. 2022), PGPR (Arp et al. 2014; Zhang et al. 2023)	$\square$	$\square$	$\square$	■
Nitrospirota (syn. Nitrospirae)					
Nitrospira lenta	Nitrification (Daims et al. 2015)	$\square$	$\square$	$\square$	■
Planctomycetota (syn. Planctomycetes)					
Lacipirellulaceae unidentified	Xylanolytic and pectinolytic (Dedysh et al. 2020)	$\square$	$\square$	$\square$	■
Phycisphaerae unidentified	Xylanolytic (Naumoff et al. 2022)	$\square$	$\square$	$\square$	■
Pirellulales unidentified	Xylanolytic and pectinolytic (Dedysh et al. 2020)	$\square$	■	$\square$	■
Caulifigura coniformis	NA	$\square$	$\square$	$\square$	■
Thermostilla marina	NA	$\square$	$\square$	$\square$	■
Verrucomicrobiota (syn. Verrucomicrobia)					
Opitutus terrae PB90-1	PGPR, phosphate solubilization, production of IAA, release of siderophores (Bünger et al. 2020)	$\square$	$\square$	■	$\square$

*IAA* Indole-3-acetic acid; *NA* not applicable; *PGPR* plant growth promoting (rhizo)bacteria

**Fig. 2:**
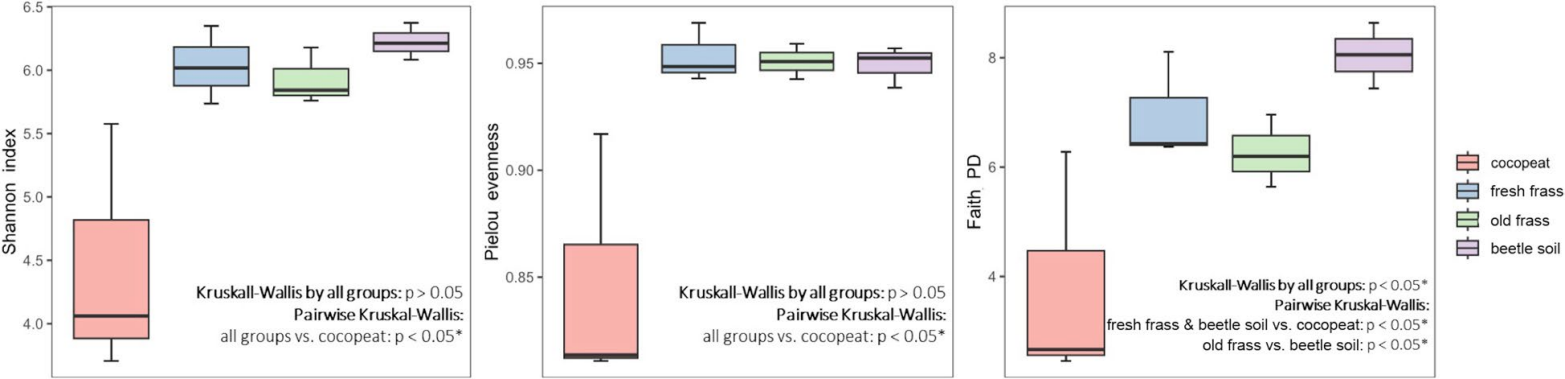
Alpha diversity indices of the microbiomes

**Fig. 3:**
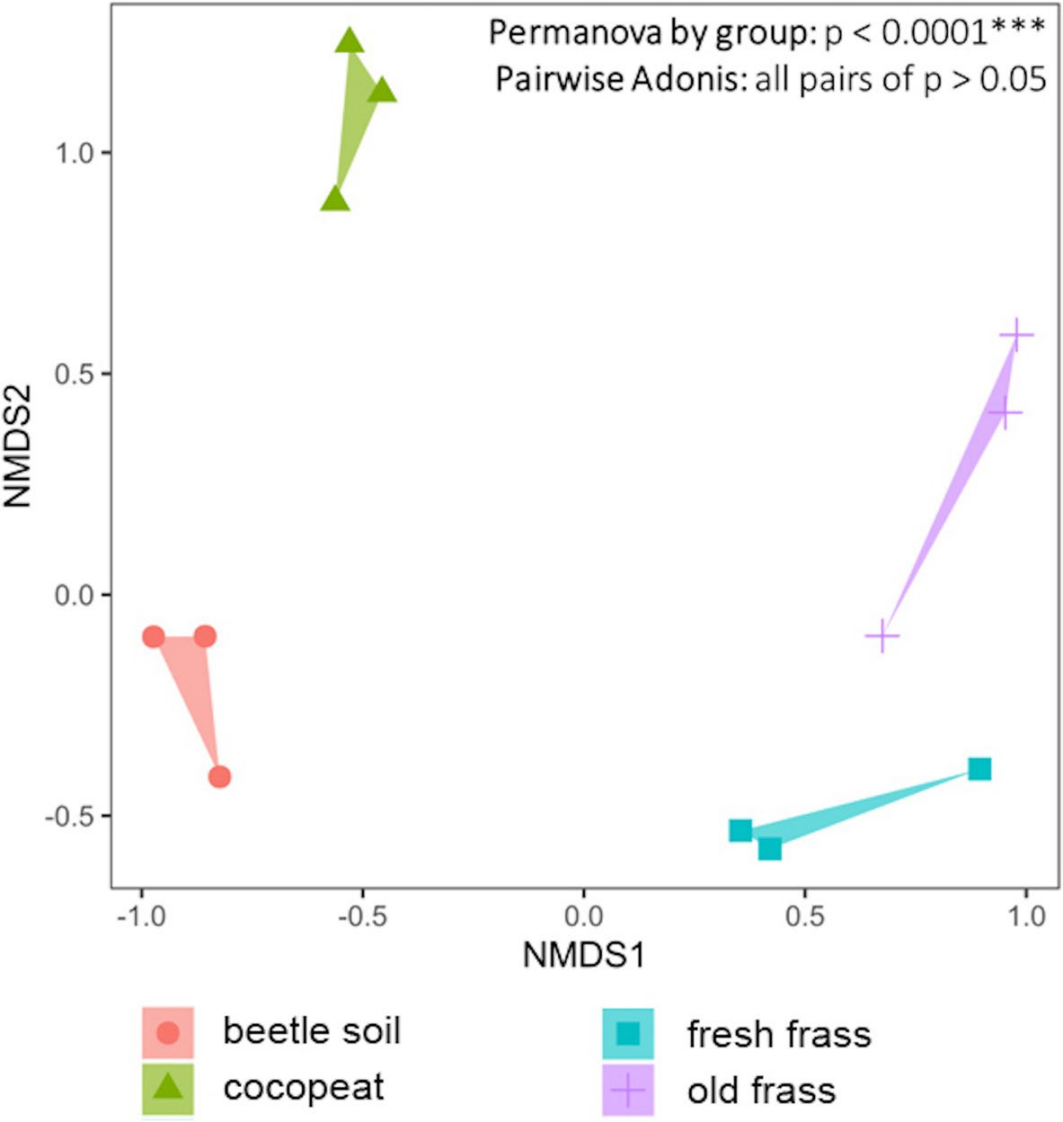
Non-metric multidimensional scaling (nMDS) plot of the microbiomes (stress = 0.109). The microbial structure is depicted using the Bray–Curtis dissimilarity matrix

The microbial species diversity of the commercial cocopeat was significantly lower than old or new frass and the lived-in substrate [“beetle soil”] (Fig. 2). The cocopeat microbiome was predominantly dominated by Firmicutes, especially unidentified Bacillaceae, a family which includes many plant-growth-promoting-bacteria (rhizobacteria) and nitrogen fixers (Fig. 1 & Table 3). By contrast, there was no specific group dominant among frass or beetle soil samples (Fig. 1), but two microbes, an unidentified Comamonadaceae and *Povalibacter uvarum* (Steroidobacterales: Steroidobacteraceae)*,* were found in all frass and beetle soil samples (Fig. 1 & Table 3). The frass and beetle soil exhibited similarly high species diversity but distinct microbiomes, containing many species PICRUSt2 analysis suggested are potentially beneficial for plants (Figs. 1, 3 & Table 3). The permanova test indicated significant differences (p < 0.0001) in microbial structures among cocopeat, beetle soil, fresh frass, and old frass. However, as the pairwise Adonis did not reveal any significant differences between groups (p > 0.05), uncertainty persists regarding which group differs significantly from the others.

**Fig. 4:**
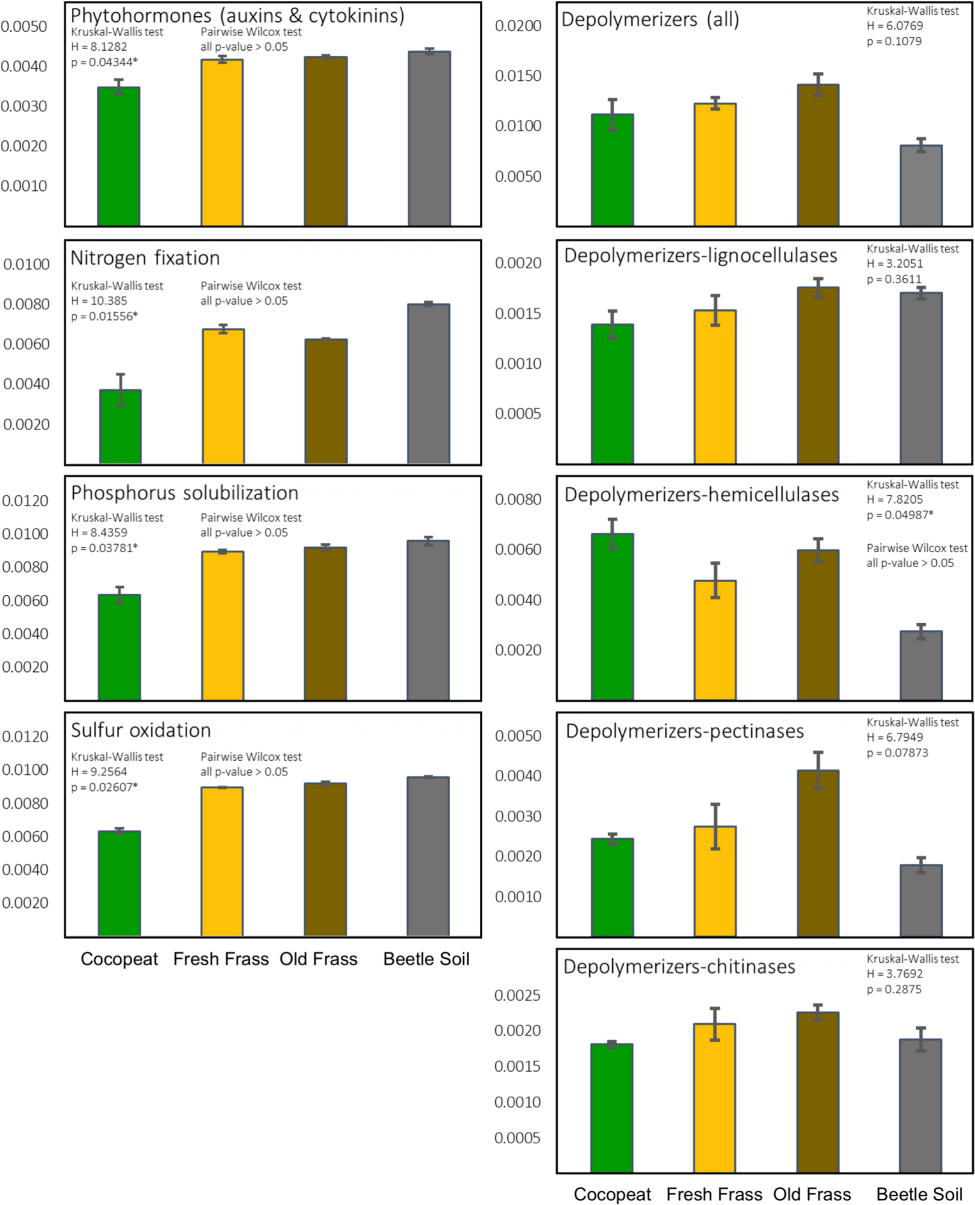
Relative abundance of the functional potentials from the microbiome. The functions were predicted using PICRUSt2, focusing on genes and enzymes related to the provision of phytohormone synthesis, nitrogen fixation, phosphorus solubilization, sulfur oxidation, plant cell wall degradation, and exoskeleton degradation

Figure 4 visually depicts comparative functional potentials along with corresponding statistical analyses for the different microbiomes’ predicted capabilities of phytohormone synthesis, nitrogen fixation, phosphorus solubilization, sulfur oxidation, and depolymerization of plant cell walls and exoskeletons. Significant differences (p < 0.05) were observed in the functions of phytohormone synthesis (auxins and cytokinins), nitrogen fixation, phosphorus solubilization, sulfur oxidation, and hemicellulose degradation between groups. However, as the pairwise Wilcox test did not reveal any significant differences between groups (p > 0.05), uncertainty persists regarding which group differs significantly from the others. In addition to the statistical results, visually, the functions of phytohormone synthesis, nitrogen fixation, phosphorus solubilization, and sulfur oxidation showed a lower relative abundance in cocopeat compared to others. Synthesis of all depolymerizers, pectinases, and chitinases was more relatively abundant in the frass (especially old frass) microbiomes than the cocopeat or beetle soil (Fig. 4). The cocopeat microbiome displayed a greater relative abundance of hemicellulase synthesis than the beetle soil.

### Plant growth tests

**Fig. 5:**
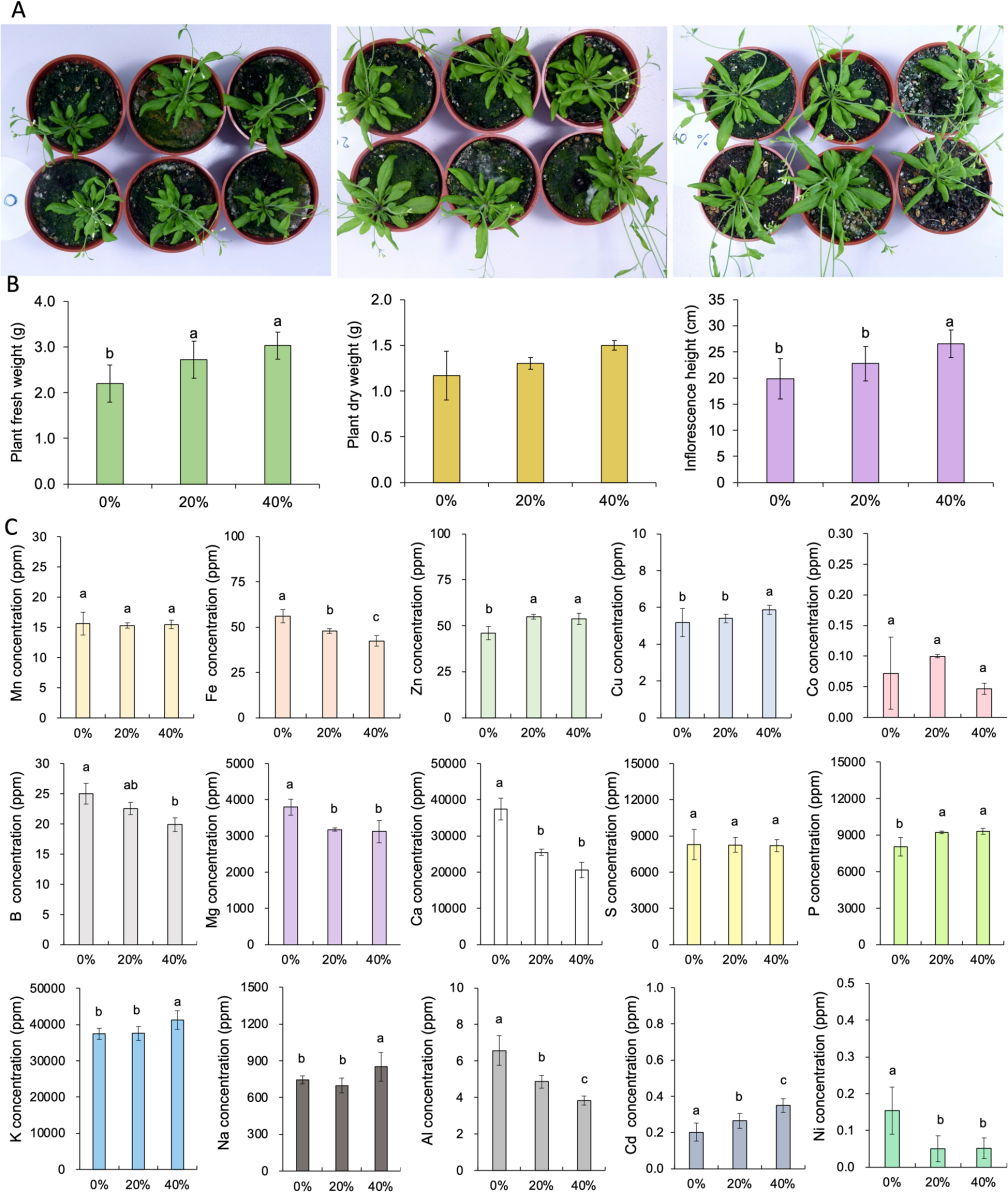
Effect of fertilization with the beetle frass on the growth of *Arabidopsis thaliana*. **A** Pictures of a representative replicate of the experiment depicting 0, 20, and 40% frass supplementation of the soil. **B** Enhanced growth of plants grown on frass-containing soil. Fresh weight (left) and dry weight (middle) of the plants and height of the inflorescence at 7 weeks (right). **C** Elemental profile of the plants established by ICP-MS. Bars with different letters are statistically significant at p < 0.05 using a Duncan test

The effect of the frass on growth of *Arabidopsis thaliana* plants was evaluated (Fig. 5a,b). The plants growing on potting soil containing frass were noticeably and statistically larger (ANOVA: F_2_ = 8.826, *p* < 0.001). In the case of the pots containing 40% frass, the biomass of the plants was significantly (*p* < 0.05) higher than in plants containing only the potting mix by 0.82 g per plant, representing an increase of 27% of their biomass (Table 4). There was no significant difference in dry weight or water content between treatments (*p* < 0.05). The frass-amended plants also started flowering earlier than plants growing on the control soil, and the latter were the last ones to start bolting. This was illustrated by the enhanced height of the inflorescence of plants grown on 40% frass at the time of harvest (30 days after planting) (ANOVA: F_2_ = 7.545, *p* < 0.01). On average, these plants were 6.7 cm taller than those grown on control soil (Table 4). Therefore, the frass had a positive effect on the growth of the plants and led to plants that grew bigger and faster than plants grown on control soil.

**Table 4:** Means ± Standard deviations for plant growth parameters for *Arabidopsis thaliana* grown in potting soil supplemented with 0, 20, or 40% *Oryctes rhinoceros* frass

% frass	Plant fresh weight (g)	Plant dry weight (g)	Water content (%)	Inflorescence height (cm after 30 days)
0%	2.2026 ± 0.4069^b^	1.1683 ± 0.2684	90.8641 ± 1.2059	19.8611 ± 3.8575^b^
20%	2.7191 ± 0.4088^a^	1.3040 ± 0.0626	91.0047 ± 0.2827	22.7625 ± 3.2826^b^
40%	3.0290 ± 0.2944^a^	1.4986 ± 0.0523	91.2163 ± 0.6709	26.5647 ± 2.6220^a^
*F*_*(2)*_* p*	8.826***			7.545**

Values followed by different superscript letters are statistically different from each other, as determined by a Duncan test with a *p*-value limit of 0.05 following one-way ANOVA, for which the *F* value (df = 2) is given with *p* values depicted as follows: • =  ≤ 0.1, * =  ≤ 0.05, ** =  ≤ 0.01 *** =  < 0.001.

With and without mixing, the pH of the potting soil and frass mixture was 5.7, suggesting the potting soil is well buffered and the frass does not have a strong buffering capacity, and excluding any effect of pH on growth. To evaluate the impact of the frass on the nutritional status of the plants, the concentration of several essential nutrients and some non-essential elements in the harvested plants were determined by ICP-MS (Fig. 5c, Supplementary Table 1). Plants grown on frass-supplemented soil had significantly increased zinc (Zn), copper (Cu), phosphorus (P), potassium (K), sodium (Na), and cadmium (Cd) concentrations. In contrast, their concentrations in iron (Fe), boron (B), magnesium (Mg), calcium (Ca), aluminum (Al), and nickel (Ni) were lower than in control plants. The most notable changes in terms of amplitude were the Ca, Al, and Ni concentrations which decreased by 45%, 42%, and 70% respectively.

Table 5 compares the elemental concentrations of the frass and the initial potting soil determined by ICP-OES. The frass had significantly higher (*p* < 0.05) concentrations of most elements, in particular aluminum, potassium, and calcium with about an order of magnitude difference. Potting soil had greater concentrations of cadmium, iron, sulfur (S), and silicon (Si).

**Table 5:** Means ± Standard deviations for elemental concentrations (mg/kg) in *Oryctes rhinoceros* frass and potting soil used in plant growth tests

Elements	Frass	Soil	*t* &* p*
Al	2022 ± 247	68 ± 6.3	− 11.18**
B	70.9 ± 14.4	43.8 ± 5.9	− 2.466•
Ca	56,619 ± 19,292	5613 ± 523	− 3.738*
Cr	5.4 ± 0.4	1.59 ± 0.4	− 8.914**
Cu	32.4 ± 3.2	36.3 ± 5.8	− 0.844
K	14,937 ± 173.4	1048 ± 420.2	− 43.21***
Mg	11,969 ± 3330	2278 ± 249	− 4.104*
Mn	261 ± 23.5	< LOD	
Na	1562 ± 86.4	287 ± 41.2	− 18.83**
P	22,667 ± 2175.8	9619 ± 331	− 8.384**
As	< LOD	< LOD	
Cd	26.31 ± 9.49	30.85 ± 5.31	− 0.723
Fe	3195 ± 65.1	4639 ± 49.9	− 30.48***
Hg	< LOD	< LOD	
Ni	< LOD	< LOD	
Pb	4.470 ± 0.56	4.617 ± 1.0	− 0.219
Rb	25.050 ± 0.78	10.767 ± 0.54	− 26.118***
S	1573 ± 10.1	4794 ± 51.7	− 106.018***
Si	< LOD	6481 ± 225.0	
Sr	126.0 ± 0.687	33.96 ± 0.814	− 149.724***
Ti	< LOD	< LOD	
Zn	96.03 ± 2.4	22.18 ± 0.16	− 52.188***
Zr	25.92 ± 5.4	19.25 ± 1.16	− 2.093•

Concentrations of the first 10 elements listed were measured via ICP-OES, the rest with pXRF. Means were compared with unpaired t-tests and the *p* values are depicted as follows: • =  ≤ 0.1, * =  ≤ 0.05, ** =  ≤ 0.01 *** =  < 0.001. LOD = limit of detection

## Discussion

One goal of composting is to lower the C:N ratio of raw materials (commonly ≥ 40) to a level closer to that of healthy soil (15–25). As the *O. rhinoceros* frass C:N ratio (22.8 ± 2.0) is within this range [and comparable to the previously reported C:N value of 14.6 ± 0.85 (Beesigamukama et al. 2022)], the frass can potentially be used directly as a soil amendment without any further composting. This was supported by the *Arabidopsis* experiments that showed higher growth with greater frass content in the soil, and the lack of evidence of phytotoxicity. Based on these results, the frass matches the definition of a biostimulant, enhancing plant growth without increasing its nutrient acquisition. It also seems more useful as a growth substrate itself, similar to how cocopeat can be used. Preliminary studies confirm that plants can germinate and grow in 100% frass, but robust experimentation would be needed to check how effective *O. rhinoceros* frass is as a growth substrate. Unknown factors include water retention and drainage in the frass that could cause nutrient leakage.

The frass microbiomes included several microbes with potential for phytohormone (auxins and cytokinins) production, nitrogen fixation, phosphorus solubilization, sulfur oxidation, and depolymerization of woody materials and chitins (Table 3 & Fig. 4). These characteristics are known to enhance plant growth and development rate (Egamberdieva et al. 2017; Richardson et al. 2009; Ranadev et al. 2023; Xu et al. 2018). In Fig. 4, the relative abundances of functional potentials from the microbiome related to phytohormones, nitrogen fixation, phosphorus solubilization, and sulfur oxidation vary significantly across different groups (p-value < 0.05), exhibiting distinct visual differences between cocopeat and frass. However, detailed differences between groups remain uncertain due to the insignificance of the pairwise Wilcoxon tests (p-value > 0.05). Possible reasons for this could include the limited sample size, which might not be large enough to detect small differences statistically, and the variability of individual microbiomes, making it challenging to distinguish true differences from background noise. Within the commonly found microbes in all frass samples as shown in Table 3 are clades with known cellulolytic, hemicellulolytic, ligninolytic, nitrogen fixation, bioremediation, or sulfur fertilization potential. These may assist in plant growth and development, thus reflecting the observed, positive effect on the growth of *Arabidopsis thaliana*.

The presence of chitinolytic microbes in the frass may help protect the plants from pathogenic fungi and nematodes while releasing nutrients from chitin that promote plant growth (Debode et al. 2016; Kisaakye et al. 2024; Shobade et al. 2024). Direct chitin supplementation of soil is known to increase both the abundance of naturally occurring chitinolytic microbes and total chitinase activity, with subsequent benefits in plant growth (Poulsen et al. 2008; Fan et al. 2022). Insect frass itself likely contains some chitin from the peritrophic membrane secreted to surround the food bolus (Chiang and Shelomi 2023). As a potential source of both chitin and chitinolytic microbes, frass could provide greater benefits than amending soil with one or the other.

Plant pathogenic microbes were not observed in this study, nor are *O. rhinoceros* known vectors of any plant pathogen to date. *O. rhinoceros* are capable of vectoring insect pathogens, however: specifically their own pathogenic bacteria, *Acinetobacter calcoaceticus* (Pseudomonadales: Moraxellaceae) (Kannan et al. 1980); pathogenic nudivirus, OrNv (Huger 2005); and their possibly pathogenic picorna-like virus, OrPV (Etebari et al. 2020). Overall, the beetle frass seems to carry beneficial bacteria while posing low risk to crops, and its formulation as a soil amendment should ideally preserve this microbiome by eschewing or minimizing pre-sterilization. Not using the frass on crops susceptible to the same bacterial infections as the palms (Arecaecae) that *O. rhinoceros* preferentially feed on would minimize any risk from transmitted pathogens. Alternatively, the ability of the beetle frass to transmit pest management viruses could mean the frass can be formulated as a combined soil amendment and biocontrol virus delivery tool, pending dedicated research on this possibility.

As an aside, the plant cell wall degrading enzymes in the microbiome likely assist the insect in fully digesting wood (Shelomi et al. 2019), and the particle size data confirms that the ingested cocopeat is significantly digested in the larval gut. Compost particle size correlates with maturity and quality (Mishra and Yadav 2022) and can affect oxygen uptake and disease suppression (Lozano et al. 2009), though the significance of the current data for the frass particles regarding compost quality is not clear.

The observed benefit of the frass to the plants is not necessarily due to its nutritional content alone. While frass was nutrient dense, the control potting soil is also nutrient-rich (Table 5) and already constitutes an optimal growth medium that does not require additional nutrients, nor did all nutrients increase in concentration in the plants when soil was replaced with frass (Fig. 5c, Supplementary Table 1). The observed changes in element concentrations in the plants were overall moderate, and some elements in plants reared in the potting soil with beetle frass supplementation showed reductions compared to the control group, such as Ca, Al, and Ni. This is striking given that the Al and Ca concentrations in the frass were over an order of magnitude greater than in the soil (Table 5). The Ca concentration in the plants grown with frass group was still within the optimal range for plant growth (Marschner 1995). As for Al and Ni, higher concentrations of them are known to inhibit and disturb the plant’s growth (Singh et al. 2017; Mustafa et al. 2023). Overall, the element concentrations measured in the soil where plants were grown suggest that supplementation with frass significantly increased most minerals in the plant, correlating with but not necessarily or solely causing overall positive effects on the plants. We hypothesize that sterilized frass lacking in beneficial microbes but retaining the same nutrients may not produce the same results as control frass.

Before now, no study had thoroughly investigated the potential of scarab beetles, especially coconut rhinoceros beetles (*O. rhinoceros*), as a plant fertilizer by combining nutritional analyses with microbiome data and plant growth assays to provide a more solid and comprehensive understanding of its applicational potential. While one previous study compared the nutritional properties of frass from *Oryctes rhinoceros* with eight other, common edible insects (Beesigamukama et al. 2022), it did not consider the effect of microbial assistance in relation to plant growth and development, nor did it conduct plant growth assays beyond germination tests. Note that this previous study on *O. rhinoceros* frass found moderate phytotoxicity of the frass as measured by reduced radical elongation in cabbage seeds reared on filter paper moistened with 10% frass compared to those on distilled water; however, the germination rate for the *O. rhinoceros* frass seeds was 93.3 ± 6.7%, equivalent to the data for black soldier fly in that study that was identified as non-phytotoxic. The current study found no evidence of phytotoxicity: by contrast, plants reared on soil amended 40% with frass outperformed soil without frass. However, Na levels were higher in such plants than the control (Fig. 5c), and sodium concentrations in the frass were significantly higher (*p* < 0.01, Table 5) than the soil, suggesting the frass may contain unideal levels of salt that may need to be mitigated or kept in mind when determining ideal usage parameters for the frass.

In conclusion, this study demonstrated beneficial impacts on plant growth and development from direct use of *O. rhinoceros* frass as a soil amendment, suggesting potential applications in agriculture as a biostimulant or growth substrate. The beetle can be said to have biocomposted the ingested cocopeat into a more valuable substance (frass), akin to other forms of composting, and this frass is a mature compost that can be used directly as a soil amendment. Use of beetle frass as soil amendment could provide additional income opportunities for beetle rearers, particularly those raising edible species. Valorizing the waste of insects into sustainable soil amendments can be both profitable and environmentally helpful as a replacement for fertilizers, a tool in permaculture or hydroponics, or restoration of degraded soils (Regina and Daniel 2021). As a side note, the authors observed that fungi could grow on beetle frass after a while if it was improperly stored. This suggests that the frass could be a potential mushroom-growing substrate once sterilized and hydrated through pasteurization.

## Supplementary Information

Additional file 1.

Additional file 2: Figure 1. Particle size distribution for undigested, commercial cocopeat (a) and *Oryctes rhinoceros* frass (b). Sync analysis type: Diff/Im g. Particle size classes are listed as volumetrically averaged diameters. The inserts show the span of particle size classes of the cocopeat feed and beetle frass. Each distribution (D#) value is the maximum size, in µm, for the #% smallest particles. Kg = Kurtois value. Mz = graphic mean. SD = standard deviation. Ski = Inclusive Graphic Skewness.

## Data Availability

The datasets generated during and/or analyzed during the current study are available in the NCBI GenBank depository (Bioproject: PRJNA1052367). The data corresponding with Fig. 5c is available as Supplementary Table 1.

## Abbreviations

ASV: Amplicon sequence variant
nMDS: Non-metric multidimensional scaling
PGPR: Plant growth promoting (rhizo)bacteria
