# Supplementary material for: Coconut rhinoceros beetle, Oryctes rhinoceros (Coleoptera: Scarabaeidae), larval frass as plant fertilizer
Source: Bot Stud. 2025 Aug 5;66:22. doi: 10.1186/s40529-025-00459-x (PMC12325837; doi:10.1186/s40529-025-00459-x)
Supplement: Supplementary file 1 — Additional file 1. [file 40529_2025_459_MOESM1_ESM.docx]

**Supplementary Table 1:** Average sample concentration (mg/L ± SD) (two repeat measurements of three replicates each containing pooled matter from six plants) for elements in plants reared in soil with 0, 20, and 40 % beetle frass supplementation. Asterisks indicate significant differences from the control based on a Duncan test at *p* < 0.05. The same data is represented visually in Figure 6c.

| Element & Analytical Line (nm) | % Beetle Frass in Soil Mix | | |
| --- | --- | --- | --- |
|  | 0% | 20% | 40% |
| Mn 257.610 | 15.63±3.25 | 15.34±0.74 | 15.5±1.25 |
| Fe 238.204 | 56.01±6.43 | 47.9±2.31* | 42.42±5.1* |
| Zn 206.200 | 45.99±5.97 | 54.85±5.3* | 53.83±7.04* |
| Cu 327.393 | 5.18±1.31 | 5.39±0.38 | 5.87±0.44* |
| Ca 317.933 | 37386.32±5168.02 | 25493.11±1491.42* | 20600.71±3702.45* |
| Mg 285.213 | 3793.47±388.14 | 3175.17±75.42* | 3124.2±532.83* |
| K 766.490 | 37454.98±2647.88 | 37521.65±3394.44 | 41284.91±4382.31* |
| Na 589.592 | 744.46±54.1 | 698.22±102.98 | 850.9±200.46* |
| P 213.617 | 8054.8±1291.25 | 9235.43±175.88* | 9301.01±413.99* |
| S 181.975 | 8276.36±2180.8 | 8258.26±1071.97 | 8202.42±870.05 |
| Cd 228.802 | 0.2±0.09 | 0.26±0.07* | 0.35±0.07* |
| B 249.677 | 24.99±3.02 | 22.55±1.74 | 19.87±1.97* |
| Co 228.616 | 0.07±0.1 | 0.1±0.0049 | 0.05±0.016 |
| Ni 231.604 | 0.15±0.11 | 0.05±0.06* | 0.05±0.048* |
| Al 396.153 | 6.57±1.41 | 4.87±0.62* | 3.83±0.445* |
