# Supplementary figures and images for: Coconut rhinoceros beetle, Oryctes rhinoceros (Coleoptera: Scarabaeidae), larval frass as plant fertilizer
Source: Bot Stud. 2025 Aug 5;66:22. doi: 10.1186/s40529-025-00459-x (PMC12325837; doi:10.1186/s40529-025-00459-x)

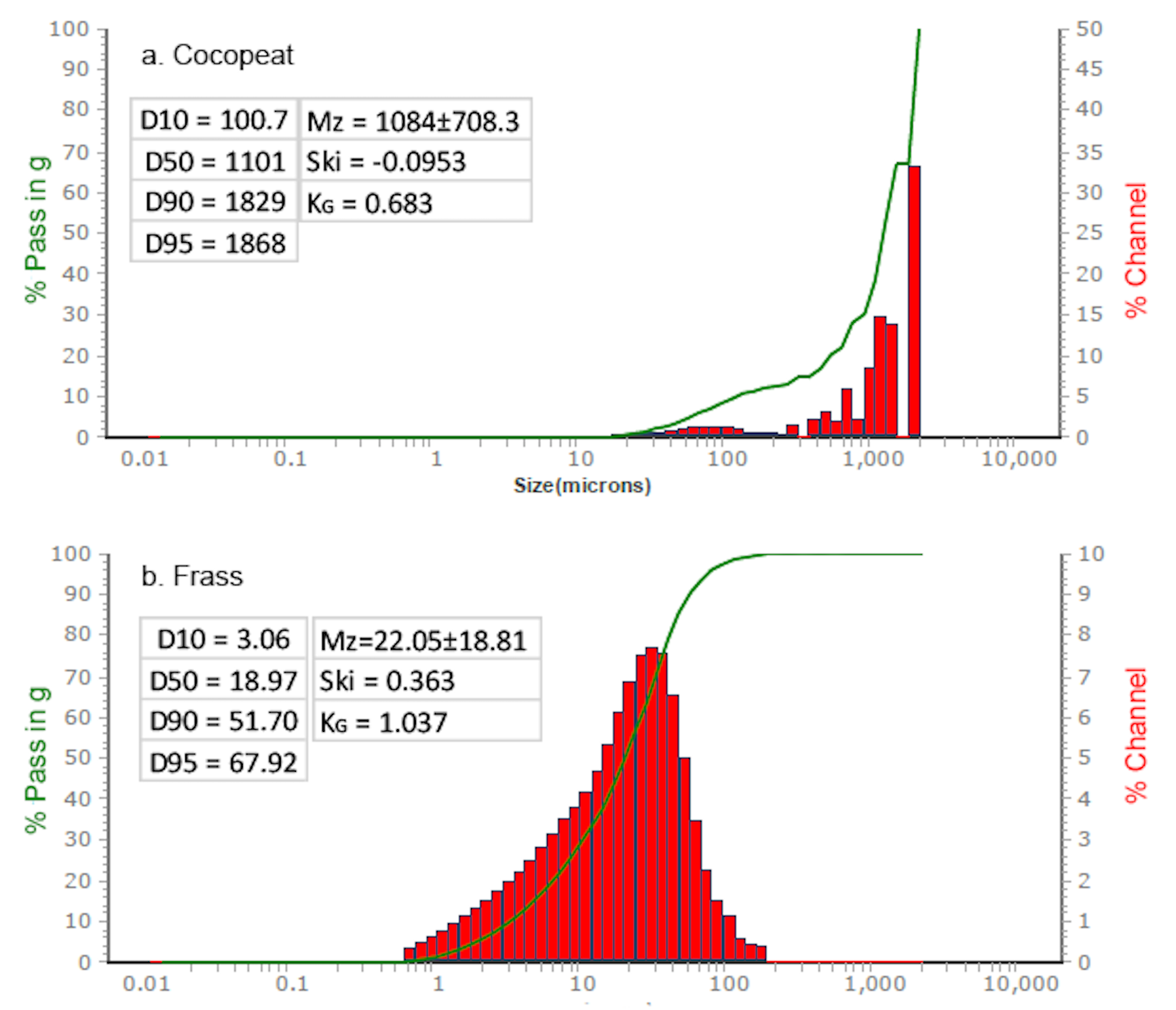

Supplement: Supplementary file 2 — Additional file 2: Figure 1. Particle size distribution for undigested, commercial cocopeat (a) and Oryctes rhinoceros frass (b). Sync analysis type: Diff/Im g. Particle size classes are listed as volumetrically averaged diameters. The inserts show the span of particle size classes of the cocopeat feed and beetle frass. Each distribution (D#) value is the maximum size, in µm, for the #% smallest particles. Kg = Kurtois value. Mz = graphic mean. SD = standard deviation. Ski = Inclusive Graphic Skewness. [file 40529_2025_459_MOESM2_ESM.png]
